# N-Acetylcysteine Dose and Treatment Duration in High-Risk Acetaminophen Ingestions Treated Within Eight Hours: A Retrospective Cohort Study

**DOI:** 10.1007/s13181-026-01129-5

**Published:** 2026-03-19

**Authors:** C. James Watson, Michael D. Simpson, Nishita Saraiya, Christopher A. Wilkosz, Michael B. Yu, Michele M. Burns, Mark J. Neavyn, Karen E. Simone, Tania D. Strout

**Affiliations:** 1 Department of Emergency Medicine, Maine Medical Center Portland, 22 Bramhall Street, Portland, ME 04102, USA; 2 Northern New England Poison Center, Portland, ME, USA; 3 Tufts University School of Medicine, Boston, MA, USA; 4 Department of Emergency Medicine, Beth Israel Deaconess Medical Center, Boston, MA, USA; 5 Massachusetts and Rhode Island Poison Center, Boston, MA, USA; 6 Harvard Medical School, Boston, MA, USA; 7 Alleghany General Hospital, Pittsburgh, PA, USA; 8 Division of Emergency Medicine, Harvard Medical, Toxicology Fellowship, Boston Children’s Hospital, Boston, MA, USA

**Keywords:** Acetaminophen, Acetylcysteine, Duration of therapy, Poisoning

## Abstract

**Introduction:**

Toxicologists may use high dose n-acetylcysteine (HD-NAC) for early-treated high-risk acetaminophen ingestions (EHRAI) given concerns over standard NAC (S-NAC) dosing’s efficacy. We utilize the novel, clinically relevant outcome of mean additional 16-hour NAC maintenance infusions (NMI) to evaluate differences in treatment duration for EHRAI patients treated with S-NAC versus HD-NAC.

**Methods:**

Retrospective multistate poison center study from 1/1/2019-7/4/2024 of patients ≥ 13-years-old who were treated with S-NAC or HD-NAC within eight hours of a high-risk acetaminophen ingestion. The primary outcome was mean additional NMI. Secondary outcomes were NAC infusion duration, hepatotoxicity, coagulopathy, transplant, and death. Sensitivity analyses evaluated for robustness of findings.

**Results:**

Of 127 included cases, 52.0% (66/127) received HD-NAC. 7.1% (9/127) had anti-peristaltic co-ingestions. HD-NAC cases received more fomepizole (23.1% versus 1.6%; difference 21.1%). Acetaminophen concentrations controlled for time since ingestion were similar (mean acetaminophen ratio for HD-NAC: 2.7, IQR: 2.3, 3.3 versus S-NAC: 2.4, IQR: 2.2, 2.7; difference – 0.3). 26.0% (33/127) received NMI solely for residual detectable serum acetaminophen. There was no difference in mean additional NMI (HD-NAC: 0.53 versus S-NAC: 0.38; *p* = 0.240). Median NAC infusion durations were equal across groups (21.0; IQR: 21.0, 37.0; difference 0.0) (*p* = 0.137). One patient per group developed hepatotoxicity; there were no transplants or deaths. Sensitivity analyses yielded similar results.

**Conclusions:**

We found no difference in mean NMI for EHRAI based on NAC dose. These findings support additional NMI as an objective, common, clinically relevant outcome for acetaminophen toxicity research.

## Introduction

### Background and Relevance

Acetaminophen overdose accounts for over 45% of acute liver failure cases in the United States and causes over 20% of single substance exposure fatalities reported to U.S. poison centers (PC) [1, 2]. Acetaminophen poisoning is treated with a standard dose of 300 milligrams/kilogram of n-acetylcysteine (NAC) given over 20- or 21-hours (S-NAC). Additional 16-hour NAC maintenance infusions (NMI) (6.25 milligrams/kilogram/hour - mg/kg/hr) are ordered in the case of liver injury (elevated transaminases, coagulopathy, encephalopathy) or persistently elevated acetaminophen concentrations [3]. When initiated within eight hours of ingestion, S-NAC should prevent hepatotoxicity (defined as aspartate or alanine aminotransferases – AST or ALT – > 1000 units/liter – U/L) [4, 5]. S-NAC dosing may be inadequate, however, for early-treated high-risk acetaminophen ingestions (EHRAI). We define EHRAI as a case with a serum acetaminophen concentration greater than the Rumack-Matthew high-risk line (e.g., 300 micrograms/milliliter - mcg/mL - at four hours post-ingestion) for whom NAC treatment was initiated within eight hours of ingestion [3, 6, 7]. Case reports and retrospective cohort studies have described negative outcomes for EHRAI cases receiving S-NAC, albeit confounded by co-ingestions, treatment errors, and unrelated pathology [8–14]. Therefore, some experts recommend increasing the dose of NAC to double, triple, or quadruple the standard maintenance infusion (12.5–25 mg/kg/hr) based on stoichiometric calculations from the presenting acetaminophen concentration [7, 15]. The use of high-dose NAC (HD-NAC) for high-risk ingestions is referenced for consideration in the 2023 U.S. and Canadian consensus statement on acetaminophen poisoning, and is recommended in the 2020 Australian and New Zealand management guidelines [3, 16].

The increased use of HD-NAC has not been associated with improved EHRAI outcomes including hepatotoxicity, transplant, or death [17–19]. Hepatotoxicity has historically been the standard outcome in acetaminophen research [4, 5, 20–24], but it is of limited clinical significance. To illustrate, an ALT of 1100 U/L versus 900 U/L does not inherently correlate with pain, quality of life, time until medical clearance, or other direct patient outcomes. Conversely, acute liver failure, need for transplant, and death are clinically relevant outcomes. However, these events are rare and impractical for modern acetaminophen research.

Compared with transplant or death, treatment duration beyond the initial 20-/21-hours is relatively common for high-risk ingestions [19]. Furthermore, in contrast to isolated hepatotoxicity, treatment duration is clinically relevant. Each additional NMI requires 16 more hours in-hospital before a patient is eligible for discharge or medical clearance for psychiatric evaluation. Given hospital overcrowding and psychiatric boarding [25], there is a benefit for healthcare systems to expedite the completion of medical treatment. Recent studies of HD-NAC found no difference in treatment duration across dosing strategies, although they were not adequately powered to detect such differences [18, 19]. It remains unknown whether NAC dose affects treatment duration for EHRAI.

### Aim and Hypothesis

Using retrospective data from two U.S. PCs, we aimed to utilize mean additional NMI as a proxy outcome evaluating for differences in NAC treatment duration for EHRAI patients receiving either S-NAC or HD-NAC. We hypothesized that HD-NAC would be associated with a similar number of additional NMI as compared to S-NAC.

## Methods

### Study Design

This was a retrospective cohort study of case records maintained by the Massachusetts and Rhode Island Poison Center and the Northern New England Poison Center (serving Maine, New Hampshire, and Vermont) between 1 January 2019 and 21 April 2025. This study was exempt by both PCs’ institutional review boards and follows STROBE guidelines [26].

### Setting

During the study period, neither PC had a formal protocol for the use of HD-NAC. HD-NAC was or was not recommended based on discussions between specialists in poison information (SPI) and the on-call toxicologist. The SPI relayed the recommendation to the bedside clinician, who had the final determination for treatment strategy. S-NAC at both PCs consisted of a three-bag regimen of 150 mg/kg over one hour, followed by 50 mg/kg over four hours (12.5 mg/kg/hr), then 100 mg/kg over 16 h (6.25 mg/kg/hr). Both PCs employed HD-NAC dosing outlined by Hendrickson, where the third dose was increased to 200 mg/kg over 16 h (12.5 mg/kg/hr), 300 mg/kg over 16 h (18.75 mg/kg/hr), or 400 mg/kg over 16 h (25 mg/kg/hr) for acetaminophen concentrations above the 300 mcg/mL, 450 mcg/mL, or 600 mcg/mL Rumack-Matthew nomogram lines, respectively [7]. Additional 16-hour NMI were ordered for persistent acetaminophen concentrations > 10 mcg/mL, resolving or insufficiently down trending AST or ALT, INR > 2.0, or persistent symptoms consistent with acetaminophen toxicity (defined by bedside clinicians, including persistent right upper quadrant abdominal tenderness or nausea and vomiting).

All clinical cases were recorded on the toxiCALL^®^ (Computer Automation Systems, Inc.; Aurora, CO) electronic medical record system. Bedside clinicians shared relevant details with the SPI who captured the clinical information and provided treatment recommendations, potentially in consultation with the on-call toxicologist. Concurrently, SPIs coded each case with searchable exposure agents, therapies, clinical effects, and medical outcomes; and then provided case details as free text. These durable records are maintained electronically in the toxiCALL^®^ database.

### Population and Selection Process

Consistent with previous PC research on high-risk acetaminophen ingestions, we queried toxiCALL^®^ for all cases during the study period coded with the screening inclusion criteria as an acute exposure to any acetaminophen generic substance code in a human ≥ 13-years-old for which NAC was administered. The abstractors reviewed the free text for each case for additional inclusion and exclusion criteria. Inclusion criteria fit the EHRAI definition: serum acetaminophen concentration collected 4–8 h post-ingestion ≥ two-times the Rumack-Matthew treatment line, with NAC initiation ≤ eight hours post-ingestion. Exclusion criteria derived from the PC record included: incompletely recorded data (missing initial acetaminophen concentration with timestamp, missing NAC protocol, missing outcome data); uncertain time of ingestion; deviations from PC-recommended treatment including premature discontinuation of NAC or pauses of NAC infusion > four hours; extracorporeal acetaminophen removal via hemodialysis; AST or ALT > 100 U/L on presentation; known diagnosis of cirrhosis. Co-ingestions were included and categorized. The case lists were subsequently de-identified of protected health information.

**Fig. 1:**
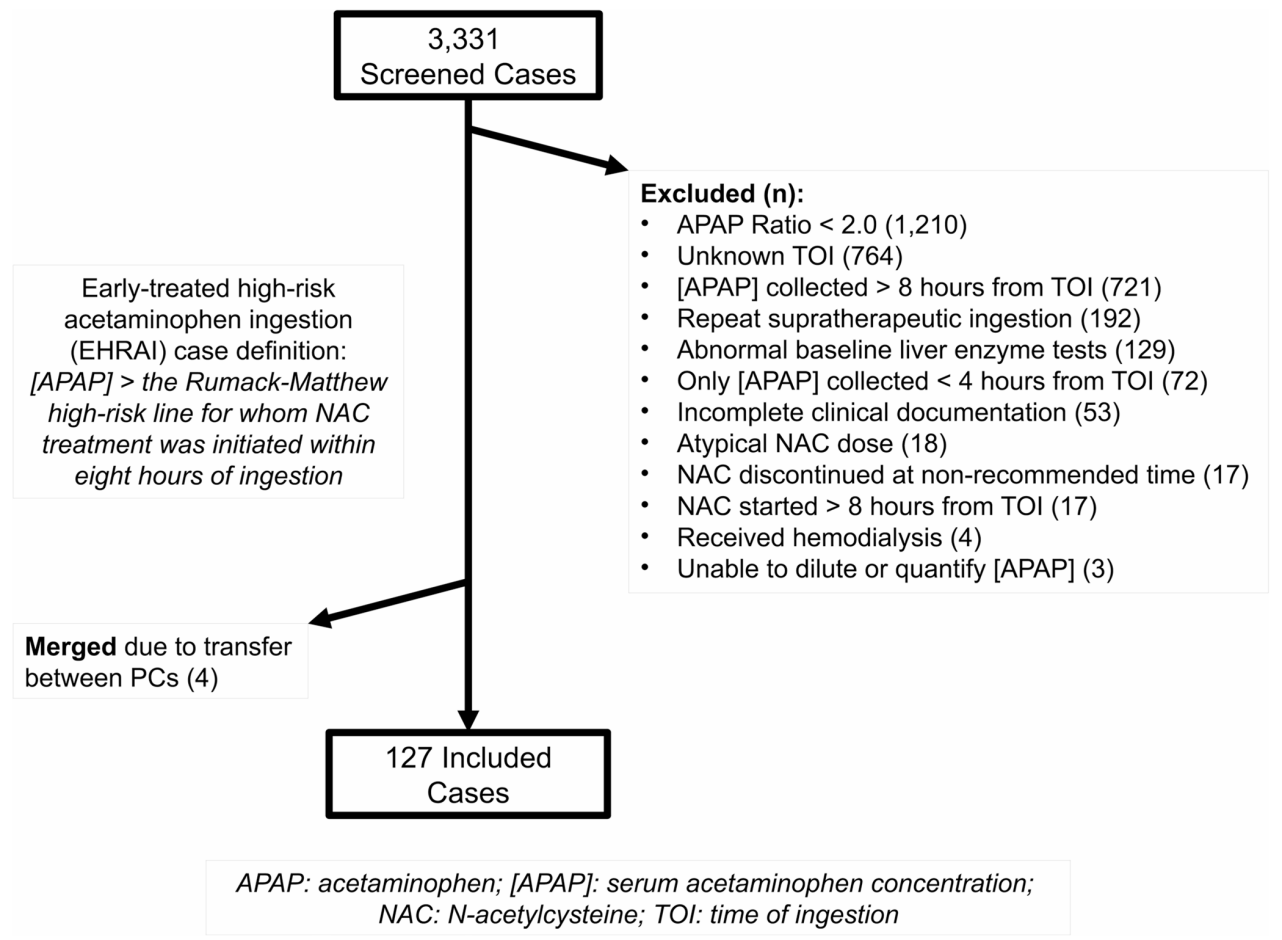
CONSORT diagram

The abstractors (MDS, NS, CAW, MY) underwent joint abstraction training. They utilized a standardized data collection sheet and an a priori defined variable dictionary. Any cases with equivocal criteria were deidentified and reviewed comprehensively by a panel of authors (CJW, MDS, NS, MY, CAW). Cases were adjudicated with predetermined rules (Fig. 1). As both PCs’ coverage areas share physical borders, cases which were transferred between regions were included only once under the receiving facility’s PC. The final de-identified case lists from each PC were maintained in REDCap^®^ (Vanderbilt University; Nashville, TN) and merged for statistical analysis via SPSS for Windows v. 28 (IBM; Armonk, NY).

### Variables and Outcome Measures

Extracted data elements included demographics (gender, age, history of liver disease, history of alcohol use disorder), substance exposure (time of ingestion, intention, reported dose, presence of co-ingestions, immediate or extended release formulation), laboratory data (initial and peak AST and ALT, INR, creatinine, pH, lactate, acetaminophen concentration, ethanol concentration), therapeutic data (time to NAC administration, time from NAC initiation to NAC termination, number of additional NMI administered following initial 21-hour course, use of gastrointestinal decontamination, use of fomepizole), clinical outcomes (time to 50% ALT peak, survival, liver transplant evaluation, liver transplant, end-stage renal disease, AST or ALT > 1000, coagulopathy, death). Gender was reported by the caller. Age was recorded by year, with those > 89-years-old kept in a single category to comply with protected health information standards. Gastrointestinal decontamination and fomepizole doses were sporadically reported, and so were not included.

Consistent with previous research, we standardized the initial acetaminophen concentration against time using the acetaminophen ratio (AR): serum acetaminophen concentration divided by the corresponding value along the Rumack-Matthew treatment line [14, 17, 19]. Notably, the Rumack-Matthew high-risk line plots an acetaminophen concentration two-times higher than the treatment line, and so we defined high-risk ingestions as having a minimum AR of 2.0. Co-ingestions were dichotomized by whether they had anti-peristaltic properties (anti-muscarinic or opioid medications). Although HD-NAC may reflect a maintenance infusion rate of 12.5 mg/kg/hr, 18.75 mg/kg/hr, or 25 mg/kg/h; these three rates reflect a stoichiometrically equivalent dose based on the serum acetaminophen concentration. These doses normalize to their respective AR, and so were not distinguished within the HD-NAC group.

The primary outcome measure was the mean number of additional NMI administered following the initial course. Additional NMI was chosen as the treatment duration proxy over two alternatives. The first, hospital length of stay, has been used previously in acetaminophen research [27, 28] but does not account for confounding delays in hospital discharge after completion of NAC therapy. These confounders include NAC completion time of day, need for post-overdose mental health screening, and boarding for psychiatric inpatient admission. Furthermore, retrospective length of stay would be an impractical measure for PC-based research, as PC documentation generally ends after medical clearance, not hospital discharge. The second considered alternative, total NAC infusion treatment duration, has also been used in previous research [18, 19, 27]. In our practice, however, NMI reflects a more clinically realistic unit of measurement. Namely, the decision point for additional treatment comes at the completion of each 16-hour NMI rather than on a continuous basis, and it would be irregular to cease treatment midinfusion. Furthermore, our PCs are uniformly precise in the documentation of each additional NMI administered, but do not primarily record the total number of infusion hours. Infusion hours would only be collectable via extrapolation from number of NMI, making them less accurate. Therefore, we selected additional NMI the primary outcome measure. Secondary outcome measures included the extrapolated NAC infusion duration (hours), hepatotoxicity (AST or ALT > 1000 U/L), coagulopathy (INR > 2.0), liver transplant, and death. Significance was set at the 5% level (*p* < 0.05).

### Sample Size

An a priori sample size estimation (2-tailed, alpha = 0.05, beta = 0.80) was conducted using G*Power (v. 3.1.9.7). Based on conventional determinations for detecting a medium-sized effect, an effect size estimate of *df* = 0.50 was used, as we are unaware of any meta-analyses on this topic in the extant literature from which a more precise effect size could be garnered. Results indicated a sample of 128 subjects would be required to detect a difference of one additional NMI between our study groups (*n* = 64 in each treatment group). We accepted an alpha of 0.05 as statistically significant. 95% confidence intervals (CI) were computed around point estimates using the exact method.

### Statistical Analysis

Data was systematically cleaned and examined for missing values. For continuous variables, data were examined for normality using Pearson’s second coefficient of skewness and visual inspection of histograms, normal P-P and Q-Q plots [29]. Additionally, the assumption of homogeneity of variances was evaluated using Levene’s test. Descriptive statistics were used to describe the characteristics of the study cohort overall as well as the study treatment groups. Measures of central tendency and dispersion were used to summarize continuous variables (e.g., age) while frequencies and percentages were used to describe categorical variables (e.g., race). Differences in mean, median, and proportion, respectively, were reported to indicate baseline differences in groups. Fisher’s exact test facilitated categorical comparison by group of additional NMI ordered solely for residual detectable serum acetaminophen.

To evaluate the effect of NAC dosing on the number of additional NMI (primary outcome) and difference in mean overall duration of additional NAC infusion in hours (secondary outcome) using the *t*-test for independent samples as well as Cohen’s *d* as a measure of effect size. The magnitude of effect sizes was interpreted using Cohen’s guidance where 0.1–0.3 represents a small effect, 0.3–0.5 an intermediate effect, and values of 0.5 and higher suggest a strong effect [30]. When data demonstrated significant skew (NAC infusion duration), comparison of the difference between medians was made using the independent samples median test (Mood’s median test) [31]. Confidence intervals around the difference between medians were calculated using the Hodges-Lehmann estimator [32] and effect size was estimated using Cramér’s *V*. Secondary outcomes were described using frequencies, percentages, and differences in proportion where appropriate.

To assess for robustness and generalizability of the findings, we performed three sensitivity analyses. The first excludes cases for which additional NMI were only ordered due to residual detectable acetaminophen, as mechanistically the dosing regimen should not affect acetaminophen clearance and therefore have no bearing on whether NAC would need to be continued to cover the acetaminophen’s ongoing metabolism. The second also excludes those receiving fomepizole – an adjuvant therapy which emerged for high-risk patients during the study period. Appreciating that many large volume ingestions result in impaired gastrointestinal motility [33] which is not assessed in this population, a third sensitivity analysis excluded those with anti-peristaltic co-ingestions that may alter absorption kinetics unrelated to NAC dose.

## Results

### Characteristics of Study Subjects

3,331 total cases were reviewed, of which 3,200 cases were excluded for not meeting inclusion and exclusion criteria (Fig. 1). Four cases reflected transfers from one PC jurisdiction to the other; these charts were merged. Ultimately, 127 cases were included. Normality assessment revealed significant skew for the calculated AR, duration of NAC infusion, and time to NAC infusion; therefore, medians and interquartile ranges are presented, nonparametric statistical testing was employed, and Cramér’s V was selected as the measure of effect size for these comparisons. Data on demographics, exposure, and treatment by NAC group are per Table 1. 48% (61/127) of patients received S-NAC, and 52.0% (66/127) received HD-NAC. Most patients were female (57.5%, 73/127), with median age 21.0 years (range 13–89 years). No patients had a documented history of liver disease and two had documented alcohol use disorder (1.6%, 2/127). Self-harm attempts accounted for 97.6% (124/127) of cases. Co-ingestions were reported in 29.9% (35/127) of all cases, and 7.1% (9/127) had an anti-peristaltic co-ingestion (two diphenhydramine, one doxylamine, three hydroxyzine, one oxycodone, two quetiapine). Exposure characteristics were similar between the two groups except that the HD-NAC group had higher initial mean acetaminophen concentration (388 mcg/mL, 95% CI: 367–408 mcg/mL vs. 311 mcg/mL, 95% CI: 295–328 mcg/mL, mean difference 77 mcg/mL, 95% CI: 50–103 mcg/mL). When controlled for time from exposure and skewness of data using median AR, however, this difference was reduced (HD-NAC: 2.7, IQR: 2.3, 3.3 vs. S-NAC: 2.4, IQR: 2.2, 2.7, difference – 0.3, 95% CI:−0.4-−0.8).

### Treatments

There was a non-significant trend toward a higher proportion of HD-NAC patients receiving gastrointestinal decontamination (13.6% vs. 6.6%, difference 7.1%, 95% CI: −4.0-18.1%). All gastrointestinal decontamination cases received activated charcoal; no other decontamination methods were documented. A higher percent of HD-NAC patients did receive fomepizole (23.1% vs. 1.6%, difference 21.1%. 95% CI: 10.1–32.6%). There was no significant difference between groups in the proportion of patients receiving additional NMI solely for residual detectable serum acetaminophen (HD-NAC: 27.3% vs. S-NAC: 24.6%, difference 2.7%, 95% CI: −12.6-17.6%).

### Main Results

Outcomes for the entire cohort are described in Table 2. There was no statistically significant difference in the primary outcome of mean number of additional NMI, with the HD-NAC and S-NAC groups respectively requiring 0.53 (95% CI: 0.33–0.73) and 0.38 (95% CI: 0.21–0.54) additional NMI (difference – 0.15, 95% CI: −0.41-0.10) (*p* = 0.240). Similarly, the median NAC infusion duration was 21.0 h for both groups (IQR: 21.0, 37.0 for both groups, difference 0.0) (*p* = 0.137). There was no significant between-group difference in the proportion of cases requiring no additional NMI (HD-NAC: 57.6% vs. S-NAC: 68.9%, difference 11.3, 95% CI: −5.5–27.0) or requiring a single additional NMI (HD-NAC: 36.4% vs. S-NAC: 26.2%, difference 10.1, 95% CI: −6.0–25.4). There was also similarity between groups in percentage of cases requiring 2–5 additional NMI. Only one patient in each group developed AST/ALT > 1,000 U/L. No patients in either group developed an INR > 2, underwent a liver transplant evaluation, or required transplant. No patients died.

### Sensitivity Analyses

A sensitivity analysis excluded cases for which the additional NMI was required solely for residual detectable serum acetaminophen (Table 3). Ninety-four total cases remained (HD-NAC: 48, S-NAC: 46), with no significant difference in median AR between groups (HD-NAC: median 2.6, IQR: 2.3, 3.3 vs. S-NAC: 2.5, IQR: 2.2, 2.7). This analysis retained eight of nine total cases with anti-peristaltic co-ingestions (four per group). Group differences included more HD-NAC patients receiving fomepizole (10.6%, 5/48 vs. 2.2%, 1/46). There was no statistically significant difference in the primary outcome of mean number of additional NMI (HD-NAC: 0.29, 95% CI: 0.06–0.52 vs. S-NAC: 0.17, 95% CI: −0.01-0.35) (*p* = 0.424). Both groups had a median NAC infusion duration of 21.0 h (IQR: 21.0, 21.0 for both groups) (*p* = 0.110). A higher proportion of S-NAC cases did not require additional NMI (HD-NAC: 79.2% vs. S-NAC: 91.3%, difference 12.1, 95% CI 02.6–26.6), whereas a higher proportion of HD-NAC cases required one additional NMI (HD-NAC: 18.8% vs. S-NAC: 2.2%, difference 16.6, 95% CI 4.0–29.9). When also excluding cases that received fomepizole (additional *n* = 6 cases removed for total *n* = 38 excluded, sample *n* = 89), the median number of additional NMI was 0.0 for both groups (IQR: 0.0, 0.0 for both groups) (*p* = 0.183) (Supplemental Data Table 1). The sensitivity analysis excluding only anti-peristaltic co-ingestions (*n* = 9 excluded, sample *n* = 118) similarly demonstrated median 0.0 additional NMI for both groups (IQR: 0.0, 1.0 each) (*p* = 0.237) with no difference in NAC infusion duration (median 21.0 h, IQR: 21.0, 37.0 for both groups) (*p* = 0.175) (Supplemental Data Table 2).

## Discussion

In this cohort of 127 EHRAI cases, there was no difference in the number of additional NMI or additional NAC treatment duration beyond 21-hours, regardless of dosing protocol. Hepatotoxicity is the traditional acetaminophen poisoning research outcome [5, 20, 24], particularly since NAC emerged as a highly effective antidote for the prevention of acute liver failure [21–23]. Simply, treatment with NAC made any outcome measuring synthetic failure, need for transplant, or death too rare for practical research. Our findings reiterate this dilemma, where only two cases developed hepatotoxicity and none developed coagulopathy, need for transplant, or death. While published cases from the early 21st century challenged the dogma that S-NAC was sufficient to prevent adverse outcomes in EHRAI, those reports rarely developed any sequelae beyond transient hepatotoxicity or elevated INR [11, 14, 34]. The EHRAI patients treated with S-NAC who required transplant or died were all complicated cases. They either had anti-peristaltic co-ingestions that altered absorption kinetics [8, 10–14], experienced a medical error leading to inappropriately early NAC termination [9, 11, 12], or their outcome was not directly attributable to acetaminophen poisoning [10, 11, 14]. In fact, of the published cases implying S-NAC failure for EHRAI, none have required transplant or died related to an isolated acetaminophen ingestion for which they were appropriately treated with S-NAC.

Existing cohort studies comparing NAC dosing strategies for EHRAI have found no consistent benefit of HD, even regarding isolated hepatotoxicity. Chiew et al. identified lower rates of hepatotoxicity in patients with AR ≥ 2.0 treated with HD-NAC, although they included patients treated up to 16 h post-ingestion in their analysis [14]. Lewis et al. found no difference in hepatotoxicity by treatment strategy for high-risk patients, including those treated less than eight hours post-ingestion [17]. Shah & Beuhler noted that simplified, single-bag HD-NAC decreased the risk of hepatotoxicity in high-risk ingestions; however early-treated patients were not included. Further, it is unknown how the comparison single-bag regimen to the three-bags of the S-NAC protocol could affect those results [35]. McElroy et al. identified no difference in hepatotoxicity between S-NAC and HD-NAC amongst those with EHRAI [18]. Moss et al. found increased odds of hepatotoxicity associated with HD-NAC versus S-NAC, but only if treated later than eight hours post-ingestion [19]. Downs et al. found that 4/44 EHRAI cases treated with S-NAC developed hepatotoxicity, albeit without documentation of co-ingestions or errors in NAC administration, and with no cases of transplantation or death [36].

Appreciating the stoichiometric arguments in favor of HD-NAC [6, 7] while acknowledging the equivocal evidence supporting its utility in EHRAI [37], we evaluated for a benefit with regards to a novel, clinically relevant outcome. That outcome is treatment duration, measured by the proxy of additional NMI required beyond the initial protocol. Within this cohort, we found no evidence that HD-NAC reduced the number of additional NMI (primary outcome) or shortened treatment duration (secondary outcome) for EHRAI patients. This persisted despite multiple sensitivity analyses designed to stress the result’s validity. This study additionally supports the existing data showing minimal risk for hepatotoxicity, coagulopathy, transplant, or death amongst EHRAI, regardless of NAC dosing regimen.

## Limitations

This study has important limitations. It is retrospective, and we cannot presume causation. Its design depends on the interpretation of medical charts coded and documented by SPIs without direct access to the patients or their hospital records. With any PC-based research, there is a risk of inaccurate or incomplete documentation that could cause erroneous case exclusion, case inclusion, and result interpretation.

Existing research in this area is sparse, limiting our ability to conduct an a priori sample size estimation based on effect size data from large trials or meta-analyses. As the body of literature grows, researchers should consider this to develop more precise estimates and reduce the possibility of type II error.

In this cohort, S-NAC patients had lower initial serum acetaminophen, suggesting a risk of confounding by potentially higher-risk patients being treated with HD-NAC. This difference is minimized, however, when accounting for time from presentation (AR) and skewness of data (non-parametric measures of central tendency and variance).

Fomepizole and HD-NAC emerged concurrently as novel treatment options for high-risk acetaminophen ingestions during the study period. Fomepizole was used more commonly in the HD-NAC group, which may threaten internal validity. Importantly, however, there remained no difference in the number of additional NMI or duration of infusion by NAC protocol even when excluding those who received fomepizole. Future prospective research or retrospective research with a large-enough sample size for multivariate analyses to control for fomepizole administration would help clarify its role in managing high-risk poisonings.

Similar yet small numbers of patients in each group received gastrointestinal decontamination. All of these cases reflect administration of activated charcoal; no cases received whole bowel irrigation or other forms of decontamination. Our records do not reliably reflect the quantity of activated charcoal ordered or consumed by each patient. Receipt of activated charcoal has been associated with decreased serum acetaminophen amongst high-risk ingestions [14], and it is unknown how our results would generalize to a population with a higher frequency of activated charcoal administration.

Overall, regarding adjuvant therapies for acetaminophen toxicity (activated charcoal and fomepizole), we note a meaningful signal in our data towards more aggressive treatment overall in the HD-NAC group. While this may suggest that the HD-NAC group was sicker, or at higher risk, than the S-NAC group, the equivalent AR calculations for both groups argues against this. We note that the S-NAC group had equivalent outcome measures despite similar initial AR and smaller proportion of adjuvant therapies.

Because both PCs utilize a 16-hour NMI, the smallest difference in treatment time reliably detected in this study was 16 h. Alternative NAC protocols (e.g. checking labs every four hours) could detect smaller differences in necessary treatment time. However, a 16-hour difference in treatment time is more clinically meaningful than those shorter durations would be.

We excluded patients with initial abnormal transaminases, and may have inadvertently excluded those with chronic alcohol overuse or other states of nutritional deficiency. We do not know if HD-NAC would be beneficial in a subpopulation with reduced glutathione stores.

The study population is limited to cases with PC consultation, and so results do not necessarily generalize to EHRAI managed without such support. While the included cases span five states including rural and urban areas with a variety of hospital types, this region is geographically isolated to the Northeast U.S. Our results may not generalize to cases treated by PCs and toxicologists practicing in other regions of the country or world, or who manage EHRAI cases differently than do our centers.

## Conclusions

In this cohort of high-risk acetaminophen ingestions treated within eight hours of ingestion, we found no difference in the need for additional 16-hour maintenance infusions between standard- and high-dose NAC regimens. Hepatotoxicity was exceedingly rare and there were no cases of coagulopathy, transplant, or death. This study demonstrates that the number of additional NAC maintenance infusions is an objective, common, and clinically relevant outcome measure that should be considered in future acetaminophen toxicity research.

## Supplementary Material

Supplementary Material

The online version contains supplementary material available at https://doi.org/10.1007/s13181-026-01129-5.

## Figures and Tables

**Table 1:** Characteristics of the study cohort

	All	S-NAC	HD-NAC

	*n* = 127	*n* = 61	*n* = 66
DEMOGRAPHICS			
Age, years, *median (range)*	21.0 (13–89)	18.0 (13–89)	22.5 (13–83)
Gender, *n (%)*			
Female	73 (57.5)	27 (44.3)	46 (69.7)
History of liver disease, *n (%)*	0 (0)	0 (0)	0 (0)
History of alcohol use disorder, *n (%)*	2 (1.6)	0 (0)	2 (3.0)
EXPOSURE			
Self-harm intention, *n (%)*	124 (97.6)	61 (100)	63 (95.5)
Co-ingestion, *n (%)*			
Yes, any	35 (29.9)	17 (29.3)	18 (30.5)
Yes, anti-peristaltic only	9 (7.1)	5 (8.2)	4 (6.1)
Extended-release formulation, *n (%)*	3 (2.4)	0 (0)	3 (4.5)
Initial [APAP], *mean (95% CI)*	351 (336–366)	311 (295–328)	388 (367–408)
Hours from TOI to [APAP], *median (IQR)*	4.5 (4.0, 5.8)	5.0 (4.0, 6.0)	4.0 (4.0, 4.8)
APAP Ratio, *median (IQR)*	2.5 (2.2, 3.0)	2.4 (2.2, 2.7)	2.7 (2.3, 3.3)
TREATMENT			
GI decontamination given, n (%)	13 (10.2)	4 (6.6)	9 (13.6)
Fomepizole given, n (%)	16 (12.7)	1 (1.6)	15 (23.1)
Hours from TOI to NAC administration, *mean (95% CI)*	5.3 (5.0-5.7)	5.4 (5.0-5.8)	5.1 (4.7–5.5)
Additional NMI ordered solely for residual [APAP], *n (%)*			
No	87 (68.5)	41 (67.2)	46 (69.7)
Yes	33 (26.0)	15 (24.6)	18 (27.3)
Unknown	7 (5.5)	5 (8.2)	2 (3.0)

*APAP* acetaminophen, *[APAP]* serum APAP concentration, *GI* gastrointestinal, *HD-NAC* High dose N-acetylcysteine, *S-NAC* Standard-dose N-acetylcysteine,*NMI* 16-hour N-acetylcysteine maintenance infusion, *TOI* time of ingestion

**Table 2:** Outcome measures of the full study cohort

	All	S-NAC	HD-NAC	Difference	*p*-Value	Effect Size

	*n* = 127	*n* = 61	*n* = 66	(95% CI)		(95% CI)
PRIMARY OUTCOME						
Number of additional NMI, *mean (95% CI)*	0.46 (0.33–0.59)	0.38 (0.21–0.54)	0.53 (0.33–0.73)	−0.15 (−0.41–0.10)	0.240^a^	−0.21 (−0.56-0.14)^b^
SECONDARY OUTCOMES						
Number of additional NMI, *n (%)*						
0	80 (63.0)	42 (68.9)	38 (57.6)	11.3 (−5.5–27.0)	–	
1	40 (31.5)	16 (26.2)	24 (36.4)	10.1 (−6.0–25.4)		
2	5 (3.9 )	2 (3.3)	3 (4.5)	2.3 (−7.2–9.6)		
3	1 (0.8)	1 (1.6)	0 (0)	1.6 (−4.0–8.7)		
4	0 (0)	0 (0)	0 (0)	–		
5	1 (0.8)	0 (0)	1 (1.5)	1.5 (−4.5–8.1)		
Duration of total NAC infusion (hours), *median* *(IQR)*	21.0 (21.0, 37.0)	21.0 (21.0, 37.0)	21.0 (21.0, 37.0)	0 (0–0)	0.137^c^	0.03 (−0.09–0.62)^d^
ALT or AST > 1,000 U/L, *n (%)*	2 (1.6)	1(1.6)	1 (1.5)	0.1 (−6.6–7.3)	–	
INR > 2, *n (%)*	0 (0)	0 (0)	0 (0)	–	–	
Transplant evaluation or performed, *n (%)*	0 (0)	0 (0)	0 (0)	–	–	
Death, *n* (%)	0 (0)	0 (0)	0 (0)	–	–	

*HD-NAC* High dose N-acetylcysteine, *S-NAC* Standard-dose N-acetylcysteine,*NMI* 16-hour N-acetylcysteine maintenance infusion.

a =comparison of means using t-test for independent samples, equal variances assumed (Levene’s test p = 0.258);

b =effect size estimated with Cohen’s d;

c =comparison of medians using independent samples median test (Mood’s median test);

d =effect size estimated with Cramér’s V

**Table 3:** Sensitivity analysis excluding those who received additional 16-hour N-acetylcysteine infusions solely for residual detectable serum acetaminophen

	All	S-NAC	HD-NAC	Difference	*p*-Value	Effect Size

	*n* = 94	*n* = 46	*n* = 48	(95% CI)		(95% CI)
CHARACTERISTICS						
Age, years, *median (range)*	20.0 (13–89)	17.0 (13–89)	22.0 (13–83)	–4.4 (−11.5–2.7)	–	–
Female gender, *n (%)*	52 (55.3)	17 (37.0)	35 (72.9)	36.0 (15.9–52.2)	–	–
Co-ingestion, *n (%)*						
Yes, any	26 (27.7)	13 (30.2)	13 (31.7)	1.2 −16.5–18.9)	–	–
Yes, anti-peristaltic only	8 (8.5)	4 (8.7)	4 (8.3)	0.4 (−12.0–13.0)	–	–
APAP Ratio, *Median (IQR)*	2.5 (2.2, 2.9)	2.5 (2.2, 2.7)	2.6 (2.3, 3.3)	−0.2 (−0.4–0.0)	–	–
GI decontamination given, *n* (%)	6 (6.4)	3 (6.5)	3 (6.3)	0.3 (−11.2-12.0)	–	–
Fomepizole given, *n* (%)	6 (6.5)	1 (2.2)	5 (10.6)	8.2 (−2.7–20.1)	–	–
Hours from TOI to NAC administration, *mean (95% CI)*	5.4 (5.1–5.8)	5.5 (5.0–6.0)	5.3 (4.9–5.8)	0.2 −0.5–0.9)	–	–
PRIMARY OUTCOME						
Number of additional NMI, *mean (95% CI)*	0.23 (0.09–0.38)	0.17 (−0.01-0.35)	0.29 (0.06–0.52)	0.12 −0.4–0.17)	0.424^a^	−0.17 (−0.57–0.24)^b^
SECONDARY OUTCOMES						
Number of additional NMI, *n (%)*						
0	80 (85.1)	42 (91.3)	38 (79.2)	12.1 −2.6–26.6)	–	–
1	10 (10.6)	1 (2.2)	9 (18.8)	16.6 (4.0–29.9)		–
2	2 (2.1)	2 (4.3)	0 (0)	4.4 (−3.7–14.5)		–
3	1 (1.1)	1 (2.2)	0 (0)	2.2 (−5.5–11.3)		–
4	0 (0)	0 (0)	0 (0)	–		–
5	1 (1.1)	0 (0)	1 (2.1)	2.1 (−5.8–10.9)		–
Duration of total NAC infusion (hours), *median (IQR)*	21.0 (21.0, 21.0)	21.0 (21.0, 21.0)	21.0 (21.0, 21.0)	0.0 (0.0–0.0)	0.110^c^	0.04 −0.09–0.73)^d^

*APAP* acetaminophen, *[APAP]* serum APAP concentration *GI* gastrointestinal, *HD-NAC* High dose N-acetylcysteine, *S-NAC* Standard-dose N-acetylcysteine,*NMI* 16-hour N-acetylcysteine maintenance infusion, *TOI time of ingestion.*

a =comparison of means with t-test for independent samples, equal variances assumed (Levene’s test p = 0.236);

b =effect size estimated with Cohen’s d;

c =comparison of medians with independent samples median test (Mood’s median test);

d =effect size estimated with Cramér’s V

## Data Availability

Data in this manuscript were previously presented at ACMT’s Annual Scientific Meeting, Washington, DC, 2024.
