# Supplementary material for: N-Acetylcysteine Dose and Treatment Duration in High-Risk Acetaminophen Ingestions Treated Within Eight Hours: A Retrospective Cohort Study
Source: J Med Toxicol. Author manuscript; Available in PMC 2026 Apr 14. (PMC13076740; doi:10.1007/s13181-026-01129-5)
Supplement: Supplementary Material [file NIHMS2158659-supplement-Supplementary_Material.docx]

**Supplemental Data Table 1.** Sensitivity analysis excluding those who received fomepizole and those who received additional 16-hour N-acetylcysteine infusions solely for residual detectable serum acetaminophen. (*N*=89).

|  | **All** | **S-NAC** | **HD-NAC** | **Difference** | ***p*-Value** | ***Effect Size*** |
| --- | --- | --- | --- | --- | --- | --- |
|  | ***n = 89*** | ***n = 46*** | ***n = 43*** | ***(95% CI)*** |  | ***(95% CI)*** |
| **CHARACTERISTICS** |  |  |  |  |  |  |
| **Age, years**, *median (range)* | 20.0 (13-89) | 17.0 (13-89) | 22.0 (13-83) | -3.0 (-7.0 – 0.0) | - | - |
|  |  |  |  |  |  |  |
| **Female gender**, *n (%)* | 50 (56.2) | 18 (39.1) | 32 (74.4) | 35.3 (14.7 – 51.9) | - | - |
|  |  |  |  |  |  |  |
| **Co-ingestion,** *n (%)* |  |  |  |  |  |  |
| Yes, any | 26 (29.2) | 13 (30.2) | 13 (35.1) | 5.0 (-15.1 – 24.8) | - | - |
| Yes, anti-peristaltic only | 6 (6.7) | 3 (6.5) | 3 (7.0) | 0.5 (-11.4 – 12.9) | - | - |
|  |  |  |  |  |  |  |
| **APAP Ratio,** *Median (IQR)* | 2.5 (2.2, 2.9) | 2.4 (2.1, 2.7) | 2.6 (2.3, 3.2) | -0.2 (-0.4 – 0.0) | - | - |
|  |  |  |  |  |  |  |
| **GI decontamination given,**  *n* (%) | 5 (5.6) | 2 (4.3) | 3 (7.0) | 0.3 (-8.5-14.7) | - | - |
|  |  |  |  |  |  |  |
| **Hours from TOI to NAC administration,** *mean (95% CI)* | 5.5 (5.2-5.8) | 5.5 (5.1-6.1) | 5.4 (5.0-5.9) | 0.1 (-0.6 – 0.8) | - | - |
| **PRIMARY OUTCOME** |  |  |  |  |  |  |
| **Number of additional NMI,** *mean (95% CI)* | 0.22 (0.08-0.37) | 0.15 (-0.01-0.32) | 0.30 (0.05-0.56) | -0.15 (-0.5– 0.15) | 0.317^a^ | -0.21 (-0.63 – 0.20)^b^ |
| **SECONDARY OUTCOMES** |  |  |  |  |  |  |
| **Number of additional NMI,** *n* *(%)* |  |  |  |  |  |  |
| 0 | 76 (85.4) | 42 (91.3) | 34 (79.1) | 12.2 (-2.8 – 27.5) | - | - |
| 1 | 10 (11.2) | 2 (4.3) | 8 (18.6) | 14.3 (0.8 – 28.6) |  | - |
| 2 | 1 (1.1) | 1 (2.2) | 0 (0) | 2.2 (-6.2 – 11.3) |  | - |
| 3 | 1 (1.1) | 1 (2.2) | 0 (0) | 2.2 (-6.2 – 11.3) |  | - |
| 4 | 0 (0) | 0 (0) | 0 (0) | - |  | - |
| 5 | 1 (1.1) | 0 (0) | 1 (2.3) | 2.3 (-5.6 – 12.1) |  | - |
|  |  |  |  |  |  |  |
| **Duration of total NAC infusion (hours),** *median (IQR)* | 21.0 (21.0, 21.0) | 21.0 (21.0, 21.0) | 21.0 (21.0, 21.0) | 0.0 (0.0 – 0.0) | 0.111^c^ | 0.31 (-0.08 – 0.77)^d^ |
| *APAP: acetaminophen; [APAP]: serum APAP concentration; GI: gastrointestinal; HD-NAC: High dose N-acetylcysteine; S-NAC: Standard-dose N-acetylcysteine, NMI: 16-hour N-acetylcysteine maintenance infusion; TOI: time of ingestion. ^a^=comparison of means with t-test for independent samples, equal variances assumed (Levene’s test p=0.236); ^b^=effect size estimated with Cohen’s d; ^c^=comparison of medians with independent samples median test (Mood’s median test); ^d^=effect size estimated with Cramér’s V.* | | | | | | |

**Supplemental Data Table 2.** Sensitivity analysis excluding cohort with anti-peristaltic co-ingestions. (*N*=118).

|  | **All** | **S-NAC** | **HD-NAC** | **Difference** | ***p*-Value** | ***Effect Size*** |
| --- | --- | --- | --- | --- | --- | --- |
|  | ***n = 118*** | ***n = 56*** | ***n = 62*** | ***(95% CI)*** |  | ***(95% CI)*** |
| **CHARACTERISTICS** |  |  |  |  |  |  |
| **Age, years**, *median (range)* | 20.0 (13-89) | 17.5 (13-89) | 22.0 (13-83) | -3.0 (-7.0 – 0.0) | - | - |
|  |  |  |  |  |  |  |
| **Female gender**, *n (%)* | 69 (58.5) | 25 (44.6) | 16 (71.0) | 26.4 (8.6 – 42.1) | - | - |
|  |  |  |  |  |  |  |
| **Co-ingestion,** *n (%)* |  |  |  |  |  |  |
| Yes, any | 27 (24.8) | 12 (22.6) | 15 (26.8) | 4.2 (-11.5 – 19.3) | - | - |
|  |  |  |  |  |  |  |
| **APAP Ratio,** *Median (IQR)* | 2.5 (2.2, 2.9) | 2.4 (2.2, 2.6) | 2.7 (2.3, 3.2) | -0.3 (-0.5 – 0.1) | - | - |
|  |  |  |  |  |  |  |
| **GI decontamination given,**  *n* (%) | 12 (10.2) | 3 (5.4) | 9 (14.5) | 9.1 (-2.3 – 20.5) | - | - |
|  |  |  |  |  |  |  |
| **Fomepizole given,** *n* (%) | 14 (12.0) | 0 (0.0) | 14 (23.0) | 23.0 (12.2 – 34.9) | - | - |
|  |  |  |  |  |  |  |
| **Hours from TOI to NAC administration,** *mean (95% CI)* | 5.3 (5.0 - 5.6) | 5.5 (5.1 - 6.0) | 4.6 (4.6 - 5.5) | 0.5 (-0.1 – 1.1) | - | - |
| **PRIMARY OUTCOME** |  |  |  |  |  |  |
| **Number of additional NMI,** *mean (95% CI)* | 0.4 (0.3-0.6) | 0.3 (0.2-0.5) | 0.5 (0.3-0.7) | -0.2 (-0.5– 0.03) | 0.08^a^ | -0.32 (-0.68 – 0.44)^b^ |
| **SECONDARY OUTCOMES** |  |  |  |  |  |  |
| **Number of additional NMI,** *n* *(%)* |  |  |  |  |  |  |
| 0 | 77 (65.3) | 41 (73.2) | 36 (58.1) | 15.1 (-2.1 – 30.9) | - | - |
| 1 | 36 (30.5) | 14 (25.0) | 22 (35.5) | 10.5 (-6.1 – 26.1) |  | - |
| 2 | 3 (2.5) | 0 (0) | 3 (4.8) | 4.8 (-2.4 – 13.2) |  | - |
| 3 | 1 (0.8) | 1 (1.8) | 0 (0) | 1.8 (-4.2 – 9.5) |  | - |
| 4 | 0 (0) | 0 (0) | 0 (0) | - |  | - |
| 5 | 1 (0.8) | 0 (0) | 1 (1.6) | 1.6 (-5.0 – 8.6) |  | - |
|  |  |  |  |  |  |  |
| **Duration of total NAC infusion (hours),** *median (IQR)* | 21.0 (21.0, 37.0) | 21.0 (21.0, 37.0) | 21.0 (21.0, 37.0) | 0.0 (0.0 – 0.0) | 0.06^c^ | 0.25 (-0.02 – 0.75)^d^ |
| *APAP: acetaminophen; [APAP]: serum APAP concentration; GI: gastrointestinal; HD-NAC: High dose N-acetylcysteine; S-NAC: Standard-dose N-acetylcysteine, NMI: 16-hour N-acetylcysteine maintenance infusion; TOI: time of ingestion. ^a^=comparison of means with t-test for independent samples, equal variances assumed (Levene’s test p=0.236); ^b^=effect size estimated with Cohen’s d; ^c^=comparison of medians with independent samples median test (Mood’s median test); ^d^=effect size estimated with Cramér’s V.* | | | | | | |
